# Deep-Learning Model for Mortality Prediction of ICU Patients with Paralytic Ileus

**DOI:** 10.3390/bioengineering11121214

**Published:** 2024-11-30

**Authors:** Martha Razo, Maryam Pishgar, William Galanter, Houshang Darabi

**Affiliations:** 1 Department of Mechanical and Industrial Engineering, University of Illinois Chicago, 942 W Taylor St., Chicago, IL 60607, USA; mrazo@uic.edu; 2 Daniel J. Epstein Department of Industrial and Systems Engineering, USC Viterbi School of Engineering, Andrus Gerontology Center, 3715 McClintock Ave, GER 240, Los Angeles, CA 90089, USA; pishgar@usc.edu; 3 Department of Medicine, University of Illinois Chicago, 942 W Taylor St., Chicago, IL 60607, USA; billg@uic.edu

**Keywords:** AI, deep learning, prediction model, healthcare, Paralytic Ileus, mortality prediction

## Abstract

Paralytic Ileus (PI) patients in the Intensive Care Unit (ICU) face a significant risk of death. Current predictive models for PI are often complex and rely on many variables, resulting in unreliable outcomes for such a serious health condition. Predicting mortality in ICU patients with PI is particularly challenging due to the vast amount of data and numerous features involved. To address this issue, a deep-learning predictive framework was developed using the Medical Information Mart for Intensive Care IV (MIMIC-IV) dataset, which includes data from 1017 ICU patients with PI. By employing SHAP (SHapley Additive exPlanations) analysis, we were able to narrow down the features to six distinct clinical lab items. The proposed framework, called DLMP (Deep Learning Model for Mortality Prediction of ICU Patients with PI), utilizes these six unique clinical lab items: Anion gap, Platelet, PTT, BUN, Total Bilirubin, and Bicarbonate, along with one demographic variable as inputs to a neural network consisting of only two neuron layers. DLMP achieved an outstanding prediction performance with an AUC score of 0.887, outperforming existing predictive models for ICU patients with PI. The DLMP framework significantly enhances the prediction of mortality for PI patients compared to traditional process mining and machine learning models. This model holds considerable potential for prognosis, enabling families to be better informed about the severity of a patient’s condition and to prepare accordingly. Furthermore, the model is valuable for research purposes and clinical trials.

## 1. Introduction

PI is an intestinal obstruction, intestinal contents fail to progress, causing abdominal pain and vomiting [1]. PI patients are at risk of mortality as high as 45% in the ICU setting [2]. PI patients who are admitted to ICU are especially at risk of dying because of the seriousness of their condition [3]. Early prediction of the outcome of patients diagnosed with PI in the ICU could be helpful for an appropriate treatment plan for patients. It is key that current research focuses on creating accurate models for predicting the mortality of ICU patients with PI to increase patients’ life span.

A limited number of existing models have been developed to predict the mortality of ICU patients diagnosed with PI. The most recent research was conducted by Fahad Shabbir Ahmed and colleagues in 2023, where they employed a machine learning framework to forecast the mortality of patients with PI by utilizing data extracted from the publicly accessible ICU database known as MIMIC III v1.4 [4]. The most favorable outcomes were achieved by the Radial Basis Function (RBF) kernel model using a Support Vector Machine (SVM), demonstrating an accuracy of 81.30% and an AUC of 0.81 when forecasting mortality among patients with PI during their hospital stay.

Another study in the field of predicting mortality among PI patients was led by Maryam Pishgar in 2022. Pishgar and her team introduced a process mining framework known as PMPI (Process Mining Model for Predicting PI Patient Mortality) [5], which is an adaptation of their earlier work focused on forecasting in-hospital mortality for ICU patients with diabetes [6]. PMPI displays comparable, if not superior, performance, achieving an Area under the ROC Curve (AUC) score of 0.82, surpassing the best results found in the existing literature.

Existing predictive models for Paralytic Ileus (PI) are complex and utilize numerous variables. Moreover, the results of the existing models are not reliable enough for a serious health condition such as PI. Predicting mortality in ICU patients with PI is particularly challenging due to the extensive amount of data and the numerous features involved.

Generally, there is no proof that deep learning outperforms SVM and PMPI methods for all applications. However, it has been shown that in certain cases, deep learning outperforms both SVM and PMPI [7,8]. In our specific application focused on mortality prediction for ICU patients with PI, we believe that deep learning performs better than SVM, particularly when managing large input datasets for building the model. This perspective aligns with findings [7], which highlight that a significant advantage of deep-learning models is their capacity to learn complex relationships within the data. Moreover, deep-learning models effectively analyze large-scale datasets. In the context of process mining, we argue that deep learning outperforms traditional methods because process mining models typically yield better results when working with sequential, time-based data [9]. However, this advantage does not hold for the specific patient data utilized in our research.

A study led by Mehdi Gheisari and colleagues analyzed 535 studies from five major research databases (ScienceDirect, Scopus, PubMed, Web of Science, and IEEE), ultimately selecting 42 (7.9%) focused on COVID-19 diagnosis. Patients with COVID-19 were identified via mobile apps using a variety of clinical, geographic, demographic, radio- logical, serological, and laboratory data. The results showed that AI methods, especially deep learning, significantly improved COVID-19 diagnosis [8].

In this paper, we focus on determining the mortality of ICU patients with PI after 24 h of admit using a deep-learning model that uses as inputs only six unique total clinical lab items, and single demographic information. The number of variables is reduced to the most significant variables for predicting PI patient mortality. The proposed framework is called DLMP (Deep Learning Model for Mortality Prediction of ICU Patients with Paralytic Ileus) and it demonstrates a significant performance for prediction.

## 2. Methodology

### 2.1. Data Source and Inclusion Criteria

**Figure 1:**
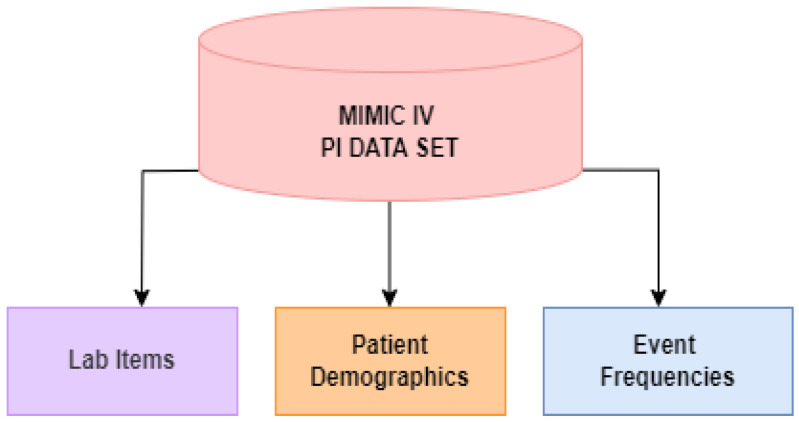
Data Source for PI Mortality Prediction.

Three distinct datasets were extracted from the MIMIC IV datasets to create the DLMP framework. MIMIC IV is a large database containing information relating to patients admitted to Beth Israel Deaconess Medical Center (BIDMC) [10]. A subset of 1067 patients is selected using the ICD-9 code for paralytic ileus, 560.1 [11] from the MIMIC-IV database. Furthermore, PI patients under 18 years of age at their first admission, and who died before 24 h of being admitted to the ICU were excluded to create a final dataset of 1017 patients. The dataset was shared by Ahmad (2023) which includes 17 lab items and 4 demographics [4]. The datasets extracted from MIMIC IV are illustrated in Figure 1. Next, we outline the individual variables present in each dataset:

Lab Items: Creatinine, Hematocrit, Hemoglobin, Platelet, PTT, Potassium, Aniongap, Blood Urea Nitrogen(BUN), Total Bilirubin, Sodium, Pt, Bicarbonate, Albumin, Lactate, White Blood count, INR, and Glucose.

Patient Demographics: Age, Gender, and Ethnicity.

**Figure 2:**
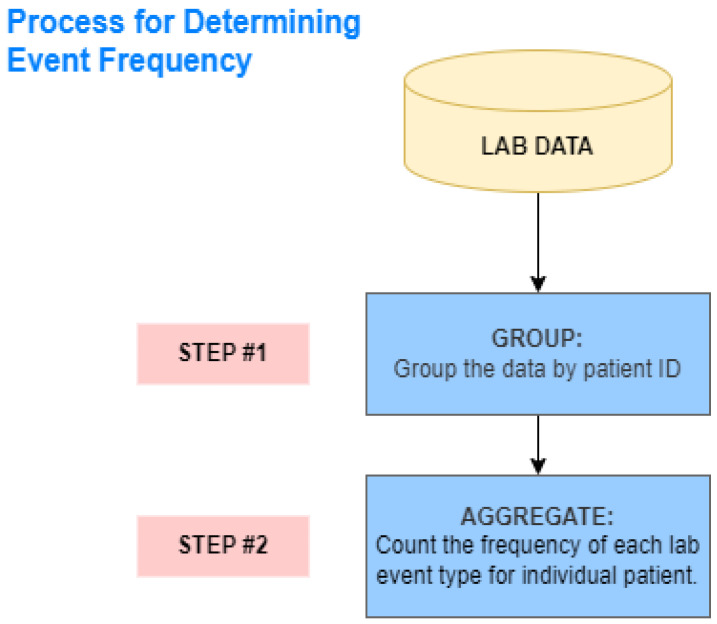
Process for determining the event frequency.

Event Frequencies: The sequence of observations on each patient is defined as an event log. Each of these observations is defined as an event. The event log used originates from the work in [5], which contains 49 unique events. Out of 49 distinct events, 3 belong to the admission type, while 10 are related to careunit activities, and represent the specific location in the ICU, patients came in and out after being admitted; CCU, CSRU, MICU, SICU, and TSICU or left the aforementioned places. Moreover, 34 of the distinct events belong to the lab measurements, 17 of them are flagged as normal and 17 of them are flagged as abnormal lab measurements. Finally, 2 of the events are the type of discharge, either death or discharge for each patient. The discharge events were excluded as these are the target events for prediction. As a result, a total of 47 event’s frequencies were used—this includes the following events: 17 lab items, abnormal lab items (ABN) (17), care unit activities (10), and ICU type upon admission (3). The frequency of each event was calculated by summing the total times an event occurred in the event log. Figure 2 shows the process for determining the event frequency.

### 2.2. Variable Importance

The number of variables was reduced using Cox-regression analysis and Kaplan–Meier survival analysis based on [4]. Furthermore, univariate analysis was created using a threshold of *p*-value = 0.02 to identify the significant variables associated with the predictor. As a result, eleven total variables were selected: five total clinical abnormal lab items (Anion gap, Platelet, PTT, BUN, and Total Bilirubin), five lab event frequencies (Bicarbonate, Total Bilirubin, BUN, Platelets, and PTT), and age. Furthermore, variable importance using Random Forest was used. As a result, the abnormal lab event of the Bicarbonate event was removed. Finally, the trade-off analysis between the number of variables and AUC score was conducted to minimize the number of variables while maintaining a high AUC score. The resulting data consist of the 11 total aforementioned variables associated with each patient.

### 2.3. Prediction of Mortality for PI Patients

**Figure 3:**
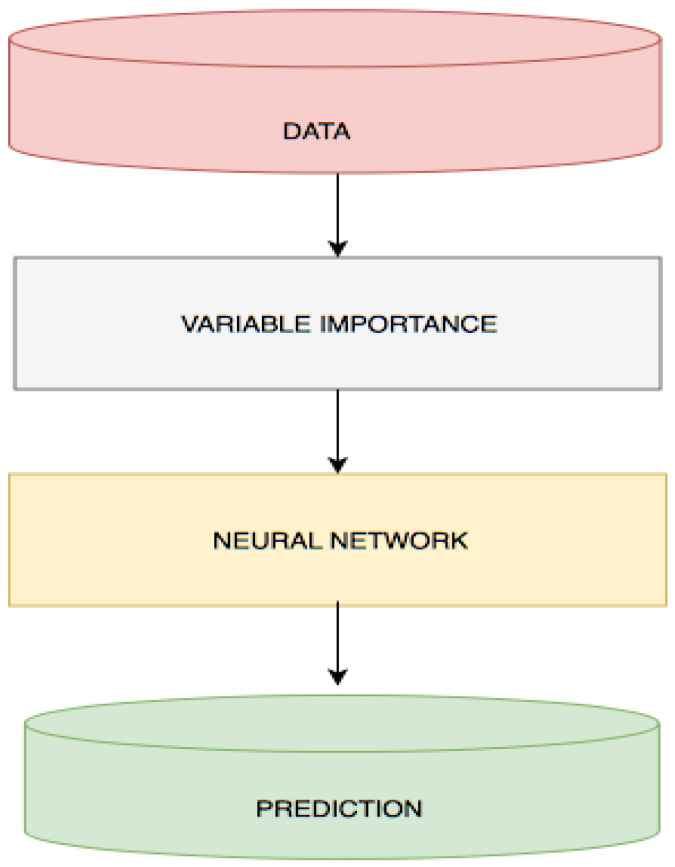
DLMP Framework.

We propose a deep-learning model for predicting the mortality of patients diagnosed with PI after 24 h of admission to the ICU. Furthermore, several machine-learning models were developed and used as baseline models for comparison of performance. The eleven variables, five total clinical abnormal lab items, the five lab event frequencies, and the one demographic (age) are fed into an NN to predict. Figure 3 illustrates the overview of the proposed model framework. The same dataset was used to build the baseline models.

### 2.4. Statistical Analysis Between Cohorts

The training and validation cohorts are compared using Chi-Square for categorical variables and two-sided *t*-tests for continuous variables. The significant level is determined based on *p* < 0.01. Descriptive statistics, model development, and statistical analysis are conducted using Python, version 3.7.

### 2.5. Variables Impacts

SHAP (SHapley Additive exPlanations) assigns each variable an importance value for a particular prediction [12]. With the SHAP analysis, we were able to interpret how each variable contributes to the prediction of mortality for ICU PI patients.

### 2.6. Ablation Analysis

**Figure 4:**
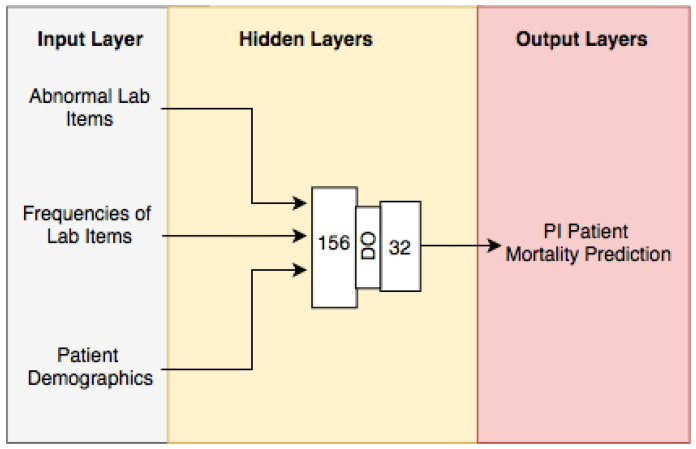
Deep -learning model architecture.

The ablation analysis was conducted for each part of the NN architecture including: the number of layers, number of neurons, learning rate, activation function, epochs, and batch size to optimize the prediction performance. Figure 4 illustrates the deep-learning model architecture.

## 3. Results

### 3.1. Cohorts Details

The description of the training and validation cohorts is presented in Table 1. The selected cohort of 1017 patients was randomly split into a training and testing split using a 67/33 ratio producing a train set of 681 patients and test set of 336 patients. Furthermore, the train set was randomly split using an 80/20 ratio to obtain a train and validation split. The validation set contained 136 patients.

The variables that were selected were the following: one demographic (Age), five total clinical abnormal lab items (Anion gap, Platelet, PTT, BUN, and Total Bilirubin), and five lab event frequencies (Bicarbonate, Total Bilirubin, BUN, Platelet, and PTT).

In terms of age, the validation cohort (61.8 years) was about the same as the training cohort (61.5 years) with a *p*-value of 0.87, which indicates that there are no significant differences between the cohorts. Furthermore, the abnormal lab items significantly differ between the cohorts with a *p*-value less than 0.05, of which the details are shown in Table 1. The frequencies of lab events in the training and validation cohorts are also significantly different between cohorts. The *p*-values values for the frequencies of lab events are less than 0.05.

### 3.2. NN Architecture Ablation Analysis

This section discusses the iterative procedure for creating the architecture of the DLMP NN.

#### 3.2.1. Layers and Neurons

The NN for the DLMP framework was initialized using the 11 input variables for the input layer, and two hidden layers of 100 and 56 neurons, respectively. The output layer consisted of one neuron with two possible outputs—one indicates that a patient died and zero shows that a patient is discharged and lives. The NN was run with an epoch of 50, batch size of 40, and learning rate of $1\times 10^{-4}$ using Adam as the optimizer. Adding and removing layers from the two hidden layers did not improve the AUC score of the NN for predicting the mortality of ICU patients with PI after 24 h of being admitted. Hence, only two layers were used for the NN final architecture. The results for changing the number of layers are shown in Table 2. Next, we found the appropriate number of neurons that would increase the model performance. The different tests are shown in Table 3. The final number architecture for the NN was two hidden layers with 156 and 32 neurons, respectively.

#### 3.2.2. Learning Rate

Increasing and decreasing the learning rate worsens the AUC score. The chosen learning rate for the NN was $5\times 10^{-4}$ using Adam as the optimizer. The tests for the learning rate are summarized in Table 4.

#### 3.2.3. Activation Function

Different combinations of activation functions were used, using the architecture (11, 156, 32, 1) with a learning rate of $5\times 10^{-4}$. The activation functions used were Relu, Sigmoid, and Tanh. All the following nine possible combinations were tested: Relu–Relu, Sigmoid–Relu, Tanh–Relu, Relu–Sigmoid, Relu–Tanh, Tanh–Sigmoid, Sigmoid–Tanh, Tanh–Tanh, and Sigmoid–Sigmoid were used for the two hidden layers and the best performance. Note, as the output is categorical, discharged, or dead, the most appropriate activation function for the output layer was softmax. In order to further improve the results, the Relu activation function was modified. The modified Relu (mod Relu) activation is defined as follows:

(1) $$f\left(z_{i}\right)=\left\{\begin{array}{cc} 0.01x+0.01 & \mathrm{for}\;x<0\\ x+0.01 & \mathrm{for}\;x\geq 0 \end{array}\right.$$

Using the mod Relu activation function, the following additional combinations were examined: Relu–mod Relu, mod Relu–Relu, and mod Relu–mod Relu. After running all 12 possible combinations of activation functions for the two hidden layers, the modified Relu activation function for both hidden layers resulted in the highest AUC. The test results are summarized in Table 5.

#### 3.2.4. Epochs & Batch Size

Finally, different epochs and batch size combinations were tested. Using the architecture (11, 156, 32, 1) with a learning rate of $5\times 10^{-4}$, and with mod Relu as the activation function for the two hidden layers. It was found that the best performance was achieved using an epoch of 150 and a batch size of 30.

The final NN for the DLMP framework architecture consisted of two hidden layers of 156 and 32 neurons respectively. The hidden layers use mod Relu as activation functions, and the output layer uses a softmax function. The NN used a learning rate of $5\times 10^{-4}$ with Adam as the learning optimizer, epochs of 150, and batch size of 30. The deep-learning model architecture for the DLMP is visualized in Figure 4.

### 3.3. SHAP Analysis

**Figure 5:**
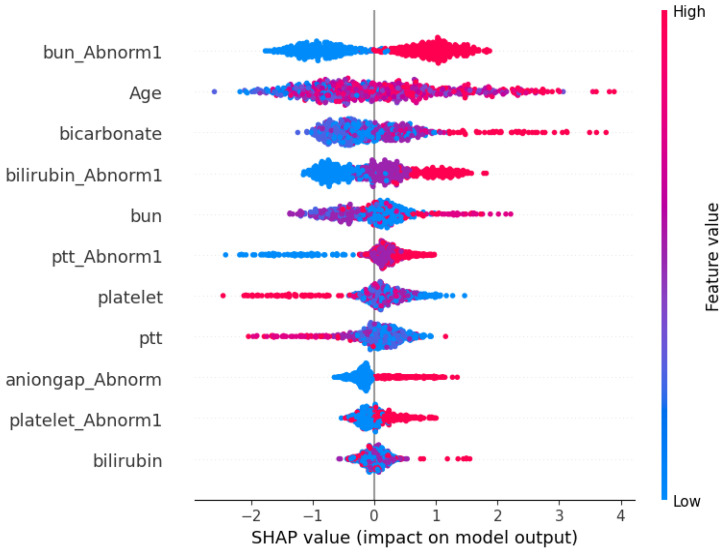
SHAPAnalysis: Variable Importance Plot.

The results of SHAP analysis are shown in Figure 5. The red color indicates a higher value of a feature, while blue indicates a lower value. High positive SHAP values indicate a higher chance of death, while lower SHAP values indicate a higher chance of survival. Based on Figure 5, the variable’s contribution to the prediction are in the following order: BUN_Abnormal1, Age, Bicarbonate, Total Bilirubin_Abnormal1, BUN, PTT_Abnormal1,Platelet, PTT, Aniongap_Abnormal1, Platelet_Abnormal1, and Total Bilirubin. Based on the SHAP values, older patients are at a higher risk of death. Furthermore, having a normal BUN lab result correlates positively with survival, indicating that patients with normal BUN values are more likely to survive. Moreover, Abnormal clinical lab results in PTT, Total Bilirubin, and Anion gap have a significant impact on the prediction of death in ICU patients with PI. Moreover, frequent Bicarbonate lab tests are associated with a higher risk of death. Conversely, an increased frequency of PTT and Platelet tests suggests a greater likelihood of survival. Lastly, the SHAP analysis suggests that the least influential predictors are the frequency of the Total Bilirubin tests and Abnormal results in Platelet and Anion gap measurements.

### 3.4. Evaluation Metrics and Proposed Model Performance

The proposed model is evaluated using the Area Under the Receiver Operating Characteristic curve (AUROC) [13] on the test cohort. The AUROC metric calculates the rate of true positive over false positive for several threshold values. DeLong’s method is used to obtain 95% Confidence Intervals (CIs) for the AUROC value [14].

The DLMP framework is built into two phases. In Phase I, the significant variables are determined. In Phase II, the optimal architecture for the NN for the DLMP is created. The DLMP framework has the best performance compared with other works and baseline machine models. The model proposed predicts with an AUC score of 0.887 and 95% CI of (0.837,0.936). The results for the DLMP prediction framework are summarized in Table 6 and compared to the developed machine learning baseline models.

## 4. Discussion

### 4.1. SHAP Analysis Interpretation Summary

From the SHAP values, it can be stated that an older patient with PI who has spent 24 h in the ICU after admission is at an increased risk of death. We also note that having an abnormal BUN lab result should raise a flag for clinicians, especially as BUN_Abnormal1 has the highest contribution to the prediction of death.

### 4.2. Existing Model Compilation Summary

In this study, we investigated a deep-learning model for predicting mortality of ICU patients with PI after 24 h of admission, in which five abnormal lab items, five frequencies of lab events, and one demographic are fed into a NN model of two neuron layers. Also, several machine learning baseline models were developed for comparison.

The DLMP framework outperformed the best results from the existing literature in terms of the AUC score proposed by [4,5]. The efficacy of our approach is demonstrated by a substantial improvement in the AUC.

The existing proposed prediction models in the literature are able to predict mortality for ICU PI patients. However, these models have some disadvantages. First, the existing models use a multitude of variables for prediction. Ahmad (2023) used a total of 17 variables, and Pishgar (2022) used 49 variables for prediction [4,5]. On the contrary, the proposed approach uses only 11 total variables which consist of only 6 total unique lab items information: Anion gap (abnormal lab event), Platelet (abnormal lab event and frequency), PTT (abnormal lab event and frequency), BUN (abnormal lab event and frequency), Total Bilirubin (abnormal lab event and frequency), and Bicarbonate (frequency). This is crucial as having fewer lab items for prediction has the potential to allow clinicians to assess a patient’s mortality with fewer lab tests. This reduces the number of resources clinicians use to make decisions regarding treatment for patients. In effect, fewer input variables have the capacity to reduce costs for the hospital or medical institution while increasing the life expectancy of ICU PI patients.

Existing models are complex in their nature. For instance, process mining has multiple steps: process discovery, creating of timed state samples, and then these timed state samples are fed into an NN. DLMP takes the data without modifications and uses only two neuron layers for prediction. The simplicity of NN allows for quick results that can be generated using any machine and can be extracted in seconds. Finally, DLMP provides prognostic insights for patients’ families. Additionally, the model can support clinical trials and research aimed at enhancing care for PI patients.

### 4.3. Study Limitations

The proposed approach has one main limitation, our model uses the MIMIC IV dataset for model development and model evaluation. Using an independent dataset from a different hospital would be optimal to test the performance of the model, which provides room for future work.

### 4.4. Optimizing Prognosis and Care with DLMP

The proposed DLMP model offers significant potential for improving estimates of prognosis compared to models that are not specific and accurate for PI. This allows families and care providers to be better informed about the severity of the illness. Beyond its prognostic capabilities, DLMP is also a valuable tool for researchers, as it can help identify when clinical adjustments are needed to optimize patient care. Moreover, its utility extends to clinical trials, where a more precise estimate of prognosis in different cohorts would allow for more precise data analysis in comparative evaluations, eventually supporting more informed decision-making in healthcare.

## 5. Conclusions

PI patients are at high risk of death when admitted to the ICU if not treated immediately. This paper demonstrates significant performance improvements in predicting the mortality of ICU patients diagnosed with PI after 24 h of being admitted. The DLMP framework predicts mortality the best with an AUC score of 0.887. The deep-learning model in the DLMP framework was fed with only five abnormal lab items, one demographic, and five lab event frequencies. DLMP is valuable for accurate prognosis, empowering medical experts to effectively communicate the severity of patients with PI in the ICU to their families. Beyond its prognostic value, DLMP is a powerful asset for advancing research and enhancing the quality of clinical trials. Future work will use another hospital’s dataset for testing the proposed model. Also, future work will partner with a medical expert to evaluate these six lab items (Bicarbonate, Total Bilirubin, BUN, Platelet, PTT, and Anion gap) with respect to PI diagnosis and mortality prediction.

## Figures and Tables

**Table 1:** Comparison of Variables in Training & Validation Cohorts.

Characteristics	Training Cohort (N = 681)	Validation Cohort (N = 136)	*p*-Value
**Outcome Variable**	N (%)		
Mortality	16.6 (113)	12.5 (17)	<0.05
**Demographics**	(Std)		
Age Mean	61.8 (15.5)	61.5 (15.9)	0.871
**Laboratory Findings**	N (%)		
Aniongap			<0.05
Aniongap_Abnormal	22.8 (155)	17.8 (24)	
Aniongap_Normal	74.4 (507)	78.7 (107)	
Aniongap_Missing	2.79 (19)	3.68 (5)	
Platelet			<0.05
Platelet_Abnormal	56.2 (383)	55.9 (76)	
Platelet_Normal	41.7 (284)	41.2 (56)	
Platelet_Missing	2.06 (14)	2.94 (4)	
PTT			<0.05
PTT_Abnormal	44.3 (302)	43.4 (59)	
PTT_Normal	43.6 (297)	41.2 (56)	
PTT_Missing	12.0 (82)	15.4 (21)	
BUN			<0.05
BUN_Abnormal	52.3 (356)	51.5 (70)	
BUN_Normal	45.8 (312)	45.6 (62)	
BUN_Missing	1.91 (13)	2.94 (4)	
Total Bilirubin			<0.05
BUN_Abnormal	40.4 (275)	48.5 (66)	
BUN_Normal	36.0 (245)	28.7 (39)	
BUN_Missing	23.6 (161)	22.8 (3)	
**Frequencies of Lab Items**	N (%)		<0.05
Bicarbonate (frequency = 0)	31.3 (213)	30.9 (42)	
Bicarbonate (frequency = 1)	26.9 (183)	30.1 (41)	
Bicarbonate (frequency = 2)	26.1 (178)	25.7 (35)	
Total Bilirubin (frequency = 0)	56.7 (386)	64.0 (87)	
Total Bilirubin (frequency = 1)	27.5 (187)	22.1 (30)	
Total Bilirubin (frequency = 2)	12.8 (87)	11.8 (16)	
BUN (frequency = 0)	41.0 (279)	40.0 (53)	
BUN (frequency = 2)	16.2 (170)	15.4 (40)	
BUN (frequency = 1)	25.0 (110)	29.4 (21)	
Platelet (frequency = 2)	28.9 (200)	27.9 (40)	
Platelet (frequency = 0)	20.1 (197)	20.6 (38)	
Platelet (frequency = 1)	29.4 (137)	29.4 (28)	
PTT (frequency = 0)	40.1 (276)	44.1 (40)	
PTT (frequency = 1)	31.1 (212)	31.2 (43)	
PTT (frequency = 2)	17.3 (118)	17.7 (24)	

**Table 2:** Test 1: Number of Layers.

Number of Layers	AUC
(11, 100, 56, 1)	0.905
(11, 100, 56, 18, 1)	0.766
(11, 100, 56, 18, 6, 1)	0.782
(11, 100, 1)	0.730
(11, 56, 1)	0.690

**Table 3:** Test 2: Number of neurons.

Number of Neurons	AUC
(11, 100, 56, 1)	0.790
(11, 130, 56, 1)	0.776
(11, 150, 56, 1)	0.769
(11, 156, 80, 1)	0.749
(11, 156, 30, 1)	0.783
(11, 156, 32, 1)	0.798
(11, 80, 56, 1)	0.753
(11, 90, 56, 1)	0.755

**Table 4:** Test 3: Learning Rate.

Learning Rate	AUC
$1\times 10^{-2}$	0.814
$1\times 10^{-3}$	0.818
$1\times 10^{-4}$	0.775
$1\times 10^{-5}$	0.520
$5\times 10^{-2}$	0.795
$5\times 10^{-3}$	0.815
$5\times 10^{-4}$	0.825
$5\times 10^{-5}$	0.687

**Table 5:** Test 4: Activation Function Combinations.

Activation Function	AUC
Relu-Relu	0.847
Sigmoid-Relu	0.817
Tanh-Relu	0.825
Relu-Sigmoid	0.837
Relu-Tanh	0.831
Tanh-Sigmoid	0.830
Sigmoid-Tanh	0.812
Tanh-Tanh	0.825
Sigmoid-Sigmoid	0.819
Relu-mod Relu	0.832
mod Relu-Relu	0.842
mod Relu-mod Relu	0.887

**Table 6:** DLMP & Machine Learning Baseline Models.

Prediction Model	AUC	AUC 95% CI
DLMP	0.887	(0.837,0.936)
Random Forest	0.830	(0.762,0.890)
Gradient Boost	0.821	(0.761,0.880)
XGBoost	0.820	(0.755,0.870)
Cat Boost	0.813	(0.753,0.872)

## Data Availability

Availability of data and materials The MIMIC-IV database which was used during the current study is publicly available [10].
